# Supplementary material for: Gender and urban–rural residency based differences in the prevalence of type-2 diabetes mellitus and its determinants among adults in Naghadeh: Results of IraPEN survey
Source: PLoS One. 2023 Mar 7;18(3):e0279872. doi: 10.1371/journal.pone.0279872 (PMC9990936; doi:10.1371/journal.pone.0279872)
Supplement: Attachment — Submitted filename: Response to reviewr (1).docx [file pone.0279872.s001.docx]

**Comments to the Author**

1. Is the manuscript technically sound, and do the data support the conclusions?

The manuscript must describe a technically sound piece of scientific research with data that supports the conclusions. Experiments must have been conducted rigorously, with appropriate controls, replication, and sample sizes. The conclusions must be drawn appropriately based on the data presented.

Reviewer #1: No

Reviewer #2: Partly

2. Has the statistical analysis been performed appropriately and rigorously?

Reviewer #1: No

Reviewer #2: No

3. Have the authors made all data underlying the findings in their manuscript fully available?

The [PLOS Data policy](http://www.plosone.org/static/policies.action#sharing) requires authors to make all data underlying the findings described in their manuscript fully available without restriction, with rare exception (please refer to the Data Availability Statement in the manuscript PDF file). The data should be provided as part of the manuscript or its supporting information, or deposited to a public repository. For example, in addition to summary statistics, the data points behind means, medians and variance measures should be available. If there are restrictions on publicly sharing data—e.g. participant privacy or use of data from a third party—those must be specified.

Reviewer #1: No

Reviewer #2: Yes

4. Is the manuscript presented in an intelligible fashion and written in standard English?

PLOS ONE does not copyedit accepted manuscripts, so the language in submitted articles must be clear, correct, and unambiguous. Any typographical or grammatical errors should be corrected at revision, so please note any specific errors here.

Reviewer #1: Yes

Reviewer #2: No

5. Review Comments to the Author

Please use the space provided to explain your answers to the questions above. You may also include additional comments for the author, including concerns about dual publication, research ethics, or publication ethics. (Please upload your review as an attachment if it exceeds 20,000 characters)

Reviewer #1: Thank you very much for your interesting manuscript! However, I have some suggestions for better scientific study.
Major comments
1. Regarding with methodology, the authors reported that they used the data from IraPEN Program in Naqadesh. The eligible criteria for the participants was set up by the census data. I understand that the authors used the secondary data for this study. You should write it clearly for using the secondary data and the study year (IraPEN program and census data) if so.

- In method section we mentioned "Using the census data" we meant, the research team included all participants who participated in IraPEN project without any sampling. In fact, 3691 people participated in IraPEN project and we included the information of all of them in secondary analysis (similar to a census).
- To reduce any ambiguity, we revised and rewrote the sentences clearly.
- Revisions: Page 7, Lines 180-182 and 188-190.
  2. The study was based on from IraPEN Program in Naqadesh. The IraPEN Program was adopted from WHO PEN for primary care actually. Therefore, you should follow the WHO STEP survey straightly for the data collection concerning anthropometric measurement and biological investigation. Currently the variable using in the study were the same as WHO STEP survey, however, the methods to measurements were not strictly follow to WHO STEP guidelines. Thus, it would make the impacts of data validity.
- IraPEN was piloted in 4 main districts of Iran. (Baft, Naghadeh, Shahrreza and Maragheh).
- The implementation of this program in the health centers of Naghadeh was carried out according to the "*Package of essential Noncommunicable diseases in Iran's primary health care system "Irapen"",* which was developed based on the WHO PEN program under the supervision of the representative office of the WHO in Iran. This instruction was provided in Persian and all health care centers in Naghadeh followed it. In fact, IraPEN program was a modified version of the STEP wise Approach to NCD Risk Factor Surveillance (STEPS) (1). In other words, the project was adjusted for Iranian setting, and that is why some differences may be seen in the measurements.
- We rewrote and added the proper reference to method section.
- Revisions:
- Pages 6 and 7: Lines 163-169; Page 8: Lines 192-194.
  3. The operational definition for smoking and alcohol consumption should be reported in details. You use another reference for it rather than WHO STEP guidelines. Could you report any special reason to do this please?

As mentioned above, all steps of IraPEN project were conducted in Naghadeh according to the "*Package of essential Noncommunicable diseases in Iran's primary health care system "Irapen"",* prepared by the Ministry of Health. We also noted that we worked on a secondary data. However, after contacting the administrative team of the study in Naghadeh, they noted that their data collection process and the instruments used for data collection were all according to the abovementioned package and the directive of the ministry. In IraPEN guideline, the practical definition of smoking and alcohol consumption was having a history of smoking and/or alcohol consumption in the past three months. So, in our present data analysis, this definition was used.

- The Package of essential Noncommunicable diseases in Iran's primary health care system "Irapen", is provided in Persian, we thought this reference could be unfamiliar to readers, so referred to a (WHO ASSIST Working Group. The Alcohol, Smoking and Substance Involvement Screening Test (ASSIST): Development, reliability and feasibility) (2).
- Please see: Pages 8- 9, Lines 214-220.

4. Concerning with fasting blood glucose investigation, you reported that the blood drawing was made twice consecutively. How did you choose the real FBS level of the participants among these two blood samples? Please describe the any special reason to do this?

As mentioned in the method section, this study was a secondary data analysis. To answer this comment, we contacted the staff of IraPEN project. They reported that only people with fasting blood glucose above 126 mg / dL were tested twice, if their fasting blood glucose in second measurement was again, 126 mg / dL or more they were diagnosed as a diabetic patient.

Revision

Please see: Page 9, Lines 228-231.
5. Regarding with plasma cholesterol levels, is it fasting or non-fasting? It would be effect on FBS level on later analysis plan.

According to the Package of essential Noncommunicable diseases in Iran's primary health care system "Irapen"(1), prepared by the Ministry of Health this test can be performed fasting or non-fasting. Moreover, lipid profiles at most change minimally in response to normal food intake in individuals in the general population (3).

We added proper sentence to method section.

Please see: Page 9, Line 235.
6. For the operational definition of T2DM, the authors defined the it (>7.0mmol/l) without using reference. You should use the WHO definition for diagnosis of DM because you used the data from IRA PEN program. In addition, you estimation may vary if you use the operational definition for diagnosis for DM (≥7.0mmol/l).

According to the Package of essential Noncommunicable diseases in Iran's primary health care system "Irapen" the definition of T2DM is Fasting plasma glucose ≥7.0mmol/l (126mg/dl). (1) Moreover, Aaccording to *"Implementation tools Package of Essential Noncommunicable (PEN) disease interventions for primary health care in low-resource settings"* the current WHO recommendations for the diagnostic criteria for diabetes and intermediate hyperglycaemia is Fasting plasma glucose ≥7.0mmol/l (126mg/dl) (4).

We provided proper reference in the main text based on WHO guideline for PEN project.

We added reference. Thanks.

Please see: Page 9, Lines 226-228.
7. The same comment for operational definition for hypertension.

According to Implementation tools Package of Essential Noncommunicable (PEN) disease interventions for primary health care in low-resource settings the current WHO recommendations for the diagnostic criteria of high blood pressure is BP ≥ 140/90 mm Hg in twice measurements. The average of two measurements was calculated.

We provided proper reference.

Please see: Page 8, Lines 212-214.
8. The authors reported that linear logistic regression in table 4 and 6. What do you mean for linear logistic regression?

We meant Simple logistic regression not linear. It was written by mistake.

We corrected it.

Please see: Page 10, Line 241.
9. What is the differences between tab 4 and 5, table 6 and 7?

- As you know, Simple logistic regression (Table 4 and 6) applies when there is a single dichotomous outcome and one independent variable while multiple logistic regression (Table 5 and 7) applies when there is a single dichotomous outcome and more than one independent variable. In fact, we used simple logistic regression to show a crude effect, and multiple logistic regression to show an adjusted effect. In other word, presenting both of these analyzes gives the reader a better insight about variables. Moreover, simple logistic regression helps to find the non-significant variables and entering them to multiple logistic regression.
- We added more details to Statistical analysis section.
- According to reviewer 2 comments we combined Table 4 and Table 5;Table 6 and Table 7.
- Please see: Page 10, Line 242-247.

10. To report the odd ratio of risk factors for developing of DM, which advanced methods was used for regression model? How did you adjust bias, confounding, mediators as you say so multiple logistic regression?

We used Multiple Forward Stepwise Logistic Regression model. In this technique, as you know, we test for the associations of potential confounders to the outcome.

The details added to Statistical analysis section.

Please see: Page 10, Line 242-247; Tables 4 and 5 legends.
11. What is the generalizability of your study?

Iran as a multi-ethnic country is composed of six major ethnic groups (5). Since previous studies have shown that the prevalence of T2DM can vary significantly depending on the ethnicity and race of individuals due to their cultural and lifestyle differences (6), so the results of this study may be mostly generalizable to the populations with Turk and Kurd ethnicities. However, it should be noted that Turkish and Kurdish ethnicities are the second and third largest ethnicities in Iran, respectively.

We added it to limitation section.

Please see: Pages 25 and 26, Lines 456-460 and 464-477.
12. You used the data from IraPEN Program in Naqadesh only. However, your title refers the prevalence of Type 2 DM and its determinants among Iranian Adults. How did you think the population in Naqadesh cover to represent all Iranian adults?

Thanks for your comment. We rewrote the title.
13. It is better to include how to calculate to have this study population?

As mentioned in the method section, this study was a secondary data analysis. So, it is not clear for us how sample size is calculated. To answer this comment, we contacted the staff of IraPEN project. They reported that no formula and/or limit is considered for sample size. Instead, based on the national guideline of the project, they planned to continue the inclusion of population into the project until the full coverage of the County population (census). When we requested the project’s data for our study, they provided us with the data of the first 3691 participants, only.
Minor comments
1. The weakness of the study is not enough yet. You should add it accordingly.

We added further weaknesses.

Please see: Page 26, Lines 464-477.

Reviewer #2: This "Gender and Urban–Rural Residency based Differences in the Prevalence of Type-2 Diabetes Mellitus and its Determinants among Iranian Adults: Results of IraPEN Survey" is a good issue for the epidemiologists and policemakers. But I have some question about this article.

1.The major weakness of this study is the representativeness. The study is using the results of IranPEN survey to explore the "Gender and Urban–Rural Residency based Differences in the Prevalence of Type-2 Diabetes Mellitus and its Determinants among Iranian Adults". Based on the survey, a total 3691 participants of 30-70 y/o were included. There were more females(56.0%) in study. Readers even didn't know about the general status in Naqadeh, ex.: the distribution of overall population by gender, age-group and race in Naqadeh. Besides, I also wonder about the representativeness because this survey was done in 2017.

We added enough information about general status in Naghadeh. We only have information about the general status in Naqadeh regarding gender. More women than men maybe due to this fact that women have a higher number of primary care visits compared to men (7).

Please see: Page 7, Lines 172-177; And Page 26 (limitations); lines 464-477.
2.The definition of rural & urban is not described clearly. What's the major economic activities in Naghadeh (agriculture, financial industry or others) ? What's the difference of this city from other A We provided definition of rural & urban in the method section.

Please see: Page 7, Lines 185-188.
3.The study included two largest ethnic groups (Trukic and Kurds). In discussion, the author talked about this mixed ethnic context can influence the prevalence of T2DM compared to other areas. I would wonder about if this two largest ethnic groups have some special characteristics from others. For example, they got higher cardiovascular diseases, thin appearance or special eating cultures. The author didn't discuss about that.

Please see: Pages 21-22, Lines 354-367.
3.Table 1 shows the baseline characteristic of participants. How about the distribution of variables compared to overall population? For example, the distribution of age-group is similar with overall of Naqadeh or not.

Despite our efforts, we could not achieve a valid data on the distribution of variables in the overall population

.We added it as a limitation to the manuscript.

Please see: Page 26 (limitations); lines 464-477.
4.Table 3 shows about the prevalence of related risk factor of T2DM by gender. I saw the low percentage of smoking status. Is that reasonable in this city?? is it comparable with the real status of Naqadeh.

In a previous study (8), the prevalence of smoking in 2016 in the northwestern provinces of Iran (Naghadeh is located in the northwestern of Iran ) was reported less than one percent in women and between 15-30 percent in men which is somewhat in accordance with our findings.

Please see: Page 23, lines 396-401.
5.Table 4 & 5 could be combined together. (the same condition with Table 6 &7.)

We combined them together.

Please see: Tables 4 and 5, Pages 15-16 AND 18-19.

I agree with the importance of the epidemiology of non-communicable diseases. We have known the prevalence & related risk factors first and make effective and feasible public policies to reduce the related co-morbidities and complications. However, the representativeness of this study is questioned.

Thanks for the comment.

Iran as a multi-ethnic country is composed of six major ethnic groups (5). Since previous studies have shown that the prevalence of T2DM can vary significantly depending on the ethnicity and race of individuals due to their cultural and lifestyle differences (6), so the results of this study may be generalizable only in two Iranian ethnicities. However, it should be noted that Turkish and Kurdish ethnicities are the second and third largest ethnicities in Iran, respectively.

We also added the representativeness issue of the study as a limitation to the limitation section.

Please see: Pages 25 and 26, Lines 456-460 and 464-477.

6. PLOS authors have the option to publish the peer review history of their article ([what does this mean?](https://journals.plos.org/plosone/s/editorial-and-peer-review-process#loc-peer-review-history)). If published, this will include your full peer review and any attached files.


If you choose “no”, your identity will remain anonymous but your review may still be made public.


**Do you want your identity to be public for this peer review?** For information about this choice, including consent withdrawal, please see our [Privacy Policy](https://www.plos.org/privacy-policy).

Reviewer #1: No

Reviewer #2: No

References

1.	A K, M N, A MH, A M, Ghanbari, A M, et al. Package of essential Noncommunicable diseases in Iran’s primary health care system “Irapen.” 1 st ed. Tehran: Mojasameh; 2017.

2.	Group WAW. The Alcohol, Smoking and Substance Involvement Screening Test (ASSIST): Development, reliability and feasibility. Addiction. 2002;97(9):1183–94.

3.	Langsted A, Freiberg JJ, Nordestgaard BG. Fasting and Nonfasting Lipid Levels. Circulation. 2008;118(20):2047–56.

4.	World Health Organisation. Implementation tools Package of Essential Noncommunicable (PEN) disease interventions for primary health care in low-resource settings. MscforumOrg. 2013;1–210.

5.	Abbasi SH, Sundin Ö, Jalali A, Soares J, Macassa G. Ethnic Differences in the Risk Factors and Severity of Coronary Artery Disease: a Patient-Based Study in Iran. Journal of Racial and Ethnic Health Disparities. 2018;5(3):623–31.

6.	Cheng YJ, Kanaya AM, Araneta MRG, Saydah SH, Kahn HS, Gregg EW, et al. Prevalence of Diabetes by Race and Ethnicity in the United States, 2011-2016. JAMA - Journal of the American Medical Association. 2019;322(24):2389–98.

7.	Bertakis KD, Azari R, Helms LJ, Callahan EJ RJ. Gender differences in the utilization of health care services. J Fam Pract. 2000;49(2):147–52.

8.	Sohrabi MR, Abbasi-Kangevari M, Kolahi AA. Current Tobacco Smoking Prevalence Among Iranian Population: A Closer Look at the STEPS Surveys. Frontiers in Public Health. 2020;8.
