# Supplementary material for: Gender and urban–rural residency based differences in the prevalence of type-2 diabetes mellitus and its determinants among adults in Naghadeh: Results of IraPEN survey
Source: PLoS One. 2023 Mar 7;18(3):e0279872. doi: 10.1371/journal.pone.0279872 (PMC9990936; doi:10.1371/journal.pone.0279872)
Supplement: Attachment — Submitted filename: Response to reviewers 2.doc [file pone.0279872.s002.doc]

Comments to the Author

1. If the authors have adequately addressed your comments raised in a previous round of review and you feel that this manuscript is now acceptable for publication, you may indicate that here to bypass the “Comments to the Author” section, enter your conflict of interest statement in the “Confidential to Editor” section, and submit your "Accept" recommendation.

Reviewer #1: All comments have been addressed

Reviewer #2: All comments have been addressed

2. Is the manuscript technically sound, and do the data support the conclusions?

The manuscript must describe a technically sound piece of scientific research with data that supports the conclusions. Experiments must have been conducted rigorously, with appropriate controls, replication, and sample sizes. The conclusions must be drawn appropriately based on the data presented.

Reviewer #1: Partly

Reviewer #2: Yes

3. Has the statistical analysis been performed appropriately and rigorously?

Reviewer #1: No

Reviewer #2: Yes

4. Have the authors made all data underlying the findings in their manuscript fully available?

The PLOS Data policy requires authors to make all data underlying the findings described in their manuscript fully available without restriction, with rare exception (please refer to the Data Availability Statement in the manuscript PDF file). The data should be provided as part of the manuscript or its supporting information, or deposited to a public repository. For example, in addition to summary statistics, the data points behind means, medians and variance measures should be available. If there are restrictions on publicly sharing data—e.g. participant privacy or use of data from a third party—those must be specified.

Reviewer #1: Yes

Reviewer #2: Yes

5. Is the manuscript presented in an intelligible fashion and written in standard English?

PLOS ONE does not copyedit accepted manuscripts, so the language in submitted articles must be clear, correct, and unambiguous. Any typographical or grammatical errors should be corrected at revision, so please note any specific errors here.

Reviewer #1: Yes

Reviewer #2: Yes

6. Review Comments to the Author

Please use the space provided to explain your answers to the questions above. You may also include additional comments for the author, including concerns about dual publication, research ethics, or publication ethics. (Please upload your review as an attachment if it exceeds 20,000 characters)

Reviewer #1: Thank you for your interesting manuscript according to the comments! However, I have some suggestions for it.

1. Table 1 and 2 should be combined and presented it as in one table.

*-We merged them.*

*Please see: Page 11, Table 1.*

2. I understand that the table 3 showed the prevalence of risk factors of the diabetes (DM) via gender and residency. However, the meaning you wrote is a little bit different if my thinking is right. Thus, be updated please.

-We rewrote it.

*Please see: Page 13.*

3. The weakness concerning the methodology of the study must be reported under the title of the limitation and strength of the study.

We reported it under the title of the limitation and strength of the study.

*Please see: Page 25, Lines* 457-462.

Reviewer #2: The author has addressed all my comments. But I still have some suggestions.

1.About the revision table 4 & 5, stepwise forward variable selection method was done. But for clinical epidemiology, the important variables should been preserved even if they didn't get significant p value, ex. smoking, alcohol...etc.

Thanks for the comment. As we performed stepwise forward regression, the insignificant variables were omitted in the next step of analysis with the hope to provide the readers with a clear presentation of the ORs. Moreover, the insignificant role of such important variables is presented in the first section of the table, where the results of the first step of regression is reported. So, the reader can get information from those variables, as well.

2.Some error wording should be corrected. For example, the "exercised" on p25 line 473. That should be "concerned" or anything else?! I advise to send this manuscript to the professional English editing.

It was corrected.

*Please see: Page 25, Line 461*.

The manuscript was edited by a professional English editor.

7. PLOS authors have the option to publish the peer review history of their article (what does this mean?). If published, this will include your full peer review and any attached files.

If you choose “no”, your identity will remain anonymous but your review may still be made public.

Do you want your identity to be public for this peer review? For information about this choice, including consent withdrawal, please see our Privacy Policy.

Reviewer #1: No

Reviewer #2: No
