# Supplementary material for: Gender and urban–rural residency based differences in the prevalence of type-2 diabetes mellitus and its determinants among adults in Naghadeh: Results of IraPEN survey
Source: PLoS One. 2023 Mar 7;18(3):e0279872. doi: 10.1371/journal.pone.0279872 (PMC9990936; doi:10.1371/journal.pone.0279872)
Supplement: Attachment — Submitted filename: Response to Reviewers.docx [file pone.0279872.s003.docx]

Reviewer #1: Thank you for your interesting manuscript. However, I have some comments yet.
1. Mercury sphygmomanometer was used for the measurement of blood pressure in the methodology. Actually, the digital blood pressure is recommended by WHO. Thus, it could be somewhat extent of variation in blood pressure and effect on analysis as I understand your availability of pressure cuff. However, you have to declare this variation and potential effect in your manuscript.

Revisions:

Thanks for your valuable comments.

Discussion; limitations; paragraph 1; lines 12-16.

Despite the recommendation of WHO to use digital sphygmomanometer, mercury sphygmomanometer was used in the IraPen study, as digital blood pressure measuring device was not available in all rural and urban health centers of the country. So, it could be somewhat extent of variation in blood pressure, which might have some potential effect on the results.

2. Fruit and vegetable consumption is one important factor for diabetes prevalence. However, this data is missing in your results.

Revisions:

Discussion; limitations; paragraph 1; lines 21-22.

Fruit and vegetable consumption is an important factor for diabetes prevalence. However, this data is missing in our results.
3. Concerning odds ratio in table 3 and 4, you presented OR of age. I understand that you reported OR of age, but it seems to be you had used the continuous data for age by logistic regression. Thus, you could add the clarification.

Response:

No, we used the categorical data for age. As Agresti recommended (1), Logistic regression, like ordinary regression, can have multiple explanatory variables, and some or all of those variables (as predictors) can be categorical, rather than quantitative. Also, an important interpretation of logistic regression model uses the odds ratio [1]. So, we can also use odds ratio as one of the ways to interpret such logistic regression analyses.

[1]	Agresti A. An introduction to categorical data analysis. 2 nd. John Wiley & Sons; 2018.

4. In addition, you used the word “cholesterol” the table 3 & 4. Generally, we could use the word “total cholesterol” instead of cholesterol. Please use the relevant word while your data is fasting lipids.

Revisions:

Tables 3 and 4 were revised, accordingly.

Reviewer #2: All comments have been addressed. I got no more comments for the authors. This draft has also finished English editing.

**Thank you**
